# Dose escalation pre-clinical trial of novel DOK7-AAV in mouse model of DOK7 congenital myasthenia

**DOI:** 10.1093/braincomms/fcaf046

**Published:** 2025-01-30

**Authors:** Judith Cossins, Imre Kozma, Claudia Canzonetta, Al Hawkins, David Beeson, Patricio Sepulveda, Yin Yao Dong

**Affiliations:** Neurosciences Group, Weatherall Institute of Molecular Medicine, John Radcliffe Hospital, University of Oxford, Oxford OX3 9DS, UK; Neurosciences Group, Weatherall Institute of Molecular Medicine, John Radcliffe Hospital, University of Oxford, Oxford OX3 9DS, UK; Amplo Biotechnology, La Jolla, CA 92037, USA; Amplo Biotechnology, La Jolla, CA 92037, USA; Neurosciences Group, Weatherall Institute of Molecular Medicine, John Radcliffe Hospital, University of Oxford, Oxford OX3 9DS, UK; Amplo Biotechnology, La Jolla, CA 92037, USA; Neurosciences Group, Weatherall Institute of Molecular Medicine, John Radcliffe Hospital, University of Oxford, Oxford OX3 9DS, UK

**Keywords:** congenital myasthenia, gene therapy, DOK7-AAV, pre-clinical trial, neuromuscular junction

## Abstract

Congenital myasthenic syndromes are a group of inherited disorders characterized by defective neuromuscular transmission and fatigable muscle weakness. Causative mutations have been identified in over 30 genes, including *DOK7*, a gene encoding a post-synaptic protein crucial in the formation and stabilization of the neuromuscular junction. Mutations in this gene are one of the leading three most prevalent causes of congenital myasthenia in diverse populations across the globe. The majority of DOK7 congenital myasthenic patients experience varying degrees of disability despite receiving optimized treatment (usually salbutamol), necessitating the development of improved therapeutic approaches. Here, we executed a dose escalation pre-clinical trial using a DOK7 congenital myasthenic syndrome mouse model to assess the efficacy of AMP-101, an innovative recombinant adeno-associated viral gene replacement therapy. This mouse model harbours a duplication in the *Dok7* gene that corresponds to the mutation most commonly found in DOK7 congenital myasthenia patients, c.1124-1127dupTGCC. The model has a much more severe phenotype than patients, and lives for only a few days. AMP-101 is based on AAVrh74 and contains human *DOK7* cDNA under the control of a muscle-restricted promoter. Three doses of AMP-101 (2 × 10^13^ vg/kg, 6 × 10^13^ vg/kg or 1 × 10^14^ vg/kg) were administered intraperitoneally at 4 days of age. We show that the two higher doses of 6 × 10^13^ vg/kg and 1 × 10^14^ vg/kg generated enlarged neuromuscular junctions and rescued the very severe phenotype of the model. Treated mice became at least as strong as wild-type littermates, as demonstrated by using an inverted screen hang test, a rotarod test and a grip strength test. EMG showed that the treated model mice had decrement of compound muscle action potential on repetitive nerve stimulation, which indicates defective signalling at the neuromuscular junction. However, male models treated with 1 × 10^14^ vg/kg showed the least decrement that was not statistically different from wild-type littermates. Western blot analysis demonstrated robust expression of DOK7 in the diaphragm and tibialis anterior muscles. These data show that AMP-101 is an effective treatment in a mouse model for DOK7 congenital myasthenia, and suggests that AMP-101 is a promising candidate to move forward to clinic trials as a gene therapy for patients.

## Teaser

Cossins *et al*. show that a novel adeno-associated viral gene therapy rescues a severe mouse model of DOK7 congenital myasthenia, making them as strong as wild-type littermates at doses comparable with adeno-associated viral therapies already used in the clinic. These data suggest that the treatment is a promising candidate for clinical trials.

## Graphical Abstract

**graphical abstract:**
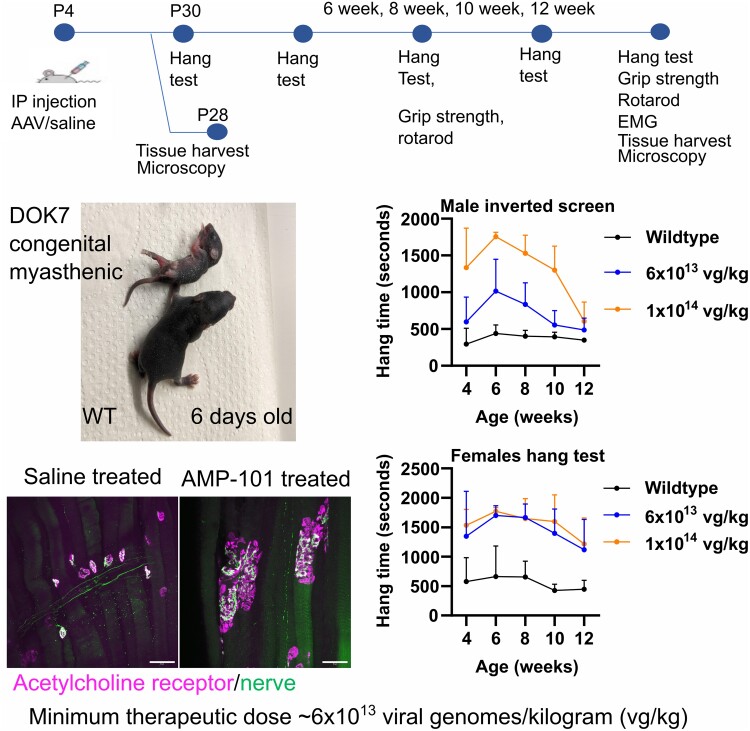

## Introduction

Congenital myasthenic syndromes (CMSs) are a group of rare inherited disorders that are characterized by fatiguable muscle weakness, which is caused by defective signalling at the neuromuscular junction (NMJ). Causative mutations in over 30 genes have been identified^1,2^ including *DOK7* that encodes the downstream of kinase 7 protein (DOK7).^3^ This protein is crucial for the dimerization and activation of muscle specific kinase (MuSK), which is a tyrosine kinase that orchestrates nicotinic acetylcholine receptor (nAChR) clustering and the formation of the NMJ.^4,5^ Patients with mutations in *DOK7* usually present during childhood with a familial limb-girdle myasthenia, and have characteristic small NMJs with a reduced number of post-synaptic folds.^3,6^

DOK7-CMS patients do not respond well to conventional CMS therapies such as the acetylcholinesterase inhibitor pyridostigmine, but ephedrine was found to be beneficial.^7-9^ However, due to the limited medical access to ephedrine, the preferred treatment is currently salbutamol (albuterol), which is as efficacious as ephedrine and has a good and extensive safety profile in children.^10-13^ Both of these medicines are β2-adrenergic receptor agonists, and although the precise therapeutic mechanism is unclear, evidence suggests that salbutamol increases both nAChR clustering *in vitro*^14^ and, importantly, increases the post-synaptic area and post-synaptic folding *in vivo*.^15^ NMJ size is thought to be important for efficient signalling and an increase should improve neurotransmission, and possibly muscle performance. However, many patients show a poor response^11^ and remain disabled even on optimized treatment. In addition, salbutamol causes tachycardia and muscle cramps in many patients,^16^ limiting the dose that can be used. Therefore, a more efficacious treatment with fewer side effects is needed.

An alternative potential treatment is gene replacement therapy whereby a functional wild-type (WT) *DOK7* gene would be introduced via a recombinant adeno-associated virus (rAAV). Arimura *et al*.^17^ highlighted the potential of this therapy when their group successfully treated a DOK7-CMS mouse model with a rAAV9 expressing human DOK7 tagged with EGFP. This knock-in mouse model is homozygous for a frameshift mutation, c.1124-1127dupTGCC, which corresponds with the most commonly observed mutation in CMS patients. These mice have a severe phenotype, gain very little weight and only survive a few days after birth. The introduced gene generated hugely enlarged NMJs and, remarkably, the mice not only thrived but also survived for a year during the follow-up period.

rAAV expressing DOK7 has also been shown to generate enlarged synapses and ameliorate symptoms in other neuromuscular diseases. In all cases, DOK7 expression was under control of ubiquitous promoters. The CMV promoter was used in models for Emery–Dreifuss muscular dystrophy^17^ and amyotrophic lateral sclerosis,^18^ and the chicken beta-actin promoter for a spinal muscular atrophy mouse model.^19^ More recently, long-term targeted expression of DOK7 in skeletal muscle of WT mice by using the tMCK promoter also generated enlarged NMJs with no evidence of adverse pathology.^20^

Using a rAAV gene transfer approach only requires a single administration, thereby circumventing the need for repeat daily dosing. However, high doses of AAV may cause liver toxicity and death, as exemplified by the death of two children with spinal muscular atrophy who received AAV9 gene therapy Zolgensma,^21^ one child who received AAV9 gene therapy PF-06939926 Duchenne muscular dystrophy gene therapy,^22^ and two patients in a recent phase 2 clinical trial of AT132, an AAV8 gene therapy in X-linked myotubular myopathy.^23^ Therefore, a lower therapeutic dose would be preferred for a safer clinical profile.

The aim of this study was to test the efficacy of three doses of a rationally optimized DOK7-AAV construct, AMP-101, in the DOK7-CMS mouse model described above (this model will be referred to as Dok-7^KI/KI^). AMP-101 is based on AAVrh74 and contains human DOK7 under control of a muscle-restricted promoter. AAVrh74 was first found in rhesus macaques, and is potentially less susceptible to treatment blockage by pre-existing serum AAV antibodies than some of the other AAV serotypes that have been isolated from humans.^24^ This, along with its ability to efficiently transduce skeletal muscle,^25-27^ and a relatively good safety profile in a DMD clinical trial^24^ make it a good gene delivery vehicle for this study.

## Materials and methods

### Dok-7 model mice

All procedures were performed in accordance with the Animals (Scientific Procedures) Act 1986 and the Home of code of practice and were approved by the University Ethical Review Panel, University of Oxford, UK. Animals were housed at 19–23°C, humidity 45–64% with 12 h light/12 h of dark, in individual ventilated caging with aspen chip bedding (dates and Eco4). They were fed with SDS RM1 food and when necessary with NutraGel, AIN-93 Bacon (LBS Serving Biotechnology). Water was RO chlorinated, and sizzle nest and tubes were included.

Dok-7^KI/KI^ homozygous for the common frameshift mutation c.1124_1127dupTGCC were generated by crossing Dok-7^+/KI^ mice with each other. Dok-7^+/KI^ animals were kindly sent to us by Prof. Yamanashi’s group, which were outbred with WT C57BL/6J mice maintained at Oxford for one generation. Eleven further generations of inbreeding were carried out prior to the start of this study.

Genotyping was performed by PCR amplification of genomic DNA obtained from tail tips at P3 using primers F1 (ATAGAGGCTGGCTTGGCAGATG) and R1 (TCCTAGCCTAACCATTGTGACTAC) followed by digestion with BamHI that recognizes the knocked-in mutation.

Prior to all behavioural test, the relevant home cages were habituated in the procedure room for 2 h.

### AMP-101

The AMP-101 expression cassette, which contains the full length human DOK7 transgene previously used by Arimura *et al*.,^17^ is driven by the striated muscle-restricted MHCK7 promoter^28^ and containing a combination of linkers, enhancers and polyA that are currently used or have previously been used in the clinic. AMP-101 was manufactured by two different companies, Viralgen and Andelyn Biosciences using HEK293 cells in suspensions followed by ultracentrifugation-based purification. All of the mice in the 3-month groups and one from the 1-month group were injected with the rAAV from Viralgen, and the virus from Andelyn Biosciences was used for the rest of the 1-month group. Virus was diluted in sterile 0.9% w/v sodium chloride in low protein binding collection tubes (Life Technologies Ltd) and stored at 4°C. The person making out the dilutions coded the tubes so that the person carrying out the rest of the project and analyses was blinded to the dose. Intraperitoneal injection of 20 μl of the AMP-101 into Dok-7^KI/KI^ mice or sterile 0.9% w/v sodium chloride into WT littermates was carried out on P4 using 0.3 ml 30 g insulin needles (VWR International Ltd). Mice were weighed every day from P3 until P30 after which they were weighed once per week.

### Mouse muscle strength

Muscle strength and fatiguability were assessed using an inverted screen hang test as previously described,^15,29^ a grip strength test (purchased from Bioseb in Vivo Research Instruments) and a rotarod (Omnitech Electronics, Inc). For the inverted screen hang test, mice were placed on a wire mesh that was inverted over a soft padded area and the length of time before the mouse fell was recorded. If the mouse held on for 10 min, it was removed. This process was carried out three times for each time point, with a 30 s rest between tests. The grip test was performed five times at each time point, with a rest of 10 s between tests, and the highest value was used for analysis. The rotarod method used an accelerating profile as follows. The mouse was placed on the rotarod that rotated at 5 rpm for 30 s, followed by a period of constant acceleration up to 40 rpm over a 5 min period. This was carried out three times for each time point, with a 5 min rest between attempts, and the highest value was used for analysis.

### Electromyography in mice

Repetitive nerve stimulation was performed as described previously.^29^ Mice were anaesthetized using inhaled isoflurane (Isoflo anaesthetic 100% w/w, Zoetis Inc). Initial induction used 1.5% isoflurane/oxygen, and this was lowered to 1.2% isoflurane/oxygen for maintenance of anaesthesia. Mouse rectal temperature was maintained between 37 and 38°C by using a heat mat underneath the mouse. The sciatic nerve was stimulated at the level of the hip. Compound muscle action potentials (CMAPs) were recorded from gastrocnemius muscles (dual bio amp/stimulator and Powerlab 4/25, AD Instruments). A train of 10 stimuli supramaximal was applied at five different frequencies of 1, 3, 5, 10 and 20 Hz. Each train was carried out three times, with a 30 s rest between trains. After the EMG procedure was complete, mice were injected sub-cutaneously with 2 mg/kg Metacam (Boehringer Ingelheim) and allowed to recover in a heated chamber before returning to the home cage.

The data were analysed using pClamp 9 (Molecular Devices).

### Immunofluorescence staining of endplate regions from mouse muscle

Quarter-diaphragms were fixed in 3% formaldehyde (TAAB Laboratory Equipment) in phosphate buffered saline (PBS) for 1 h at RT. Tissue was washed 3× with PBS and permeabilized for 1 h with 0.3% Triton-X in PBS. Tissue was washed again and blocked with 3% bovine serum albumin (BSA) in PBS for 1 h at RT. Muscle was washed in PBS and incubated overnight at 4°C in PBS containing 3% BSA, rabbit anti-neurofilament heavy chain antibody (Abcam plc, 1:1000) and rabbit anti-synaptophysin Ab-4 (Fisher Scientific, 1:100). Samples were washed extensively with PBS. nAChRs were visualized by incubating muscle with 594-alexafluor-tagged α-bungarotoxin (594-α-BuTx; Invitrogen) diluted 1:100 for 1 h at room temperature. Neurofilament and synaptophysin staining was visualized using 488-Alexafluor goat anti rabbit secondary antibody (Fisher Scientific, 1:1000). The tissue was mounted onto microscopy slides using Slowfade Diamond Antifade Mountant (Invitrogen), and z-stacks of NMJs were captured using a Zeiss 900 confocal microscope at 250× magnification. Maximum projection images were generated, and the size of the area staining positive for nAChR or neurofilament and synaptophysin was measured using ImageJ.

### Western blot

#### Tissue preparation

A total of 250 μl ice-cold extraction buffer (50 mM Tris-HCl pH 7.5, 1% Triton X-100 and 500 mM NaCl) supplemented with protease inhibitor cocktail (Sigma, P8340) was added to 2 ml tubes containing Precellys Steel beads (2.8 mm ‘reinforced’ 2 ml tubes, pre-filled with steel beads purchased from VWR International Ltd, cat. no. 432-0142). Tubes were kept on ice. Muscle samples were placed into the tubes where they were allowed to thaw on ice. Samples were homogenized using a Precellys 24 lysis homogenizer (Bertin instruments) at 4°C at 6500 rpm using a 30-5 s program twice. Samples were then rotated for 1 h at 4°C. The lysate was transferred to clean microcentrifuge tubes that were centrifuged at 13 000 rpm for 10 min at 4°C to pellet cell debris. Clarified lysate was transferred to clean microcentrifuge tubes. Samples were diluted typically 1:10, or sometimes 1:20, for protein concentration quantification using a BCA protein assay kit (Fisher Scientific UK Ltd, cat. no. 10678484).

For a positive control, HEK293T cells were transfected with the mammalian expression vector pcDNA3.1 Hygro(+) containing human *DOK7* cDNA using polyethylenimine. For a negative control, empty vector was used for the transfection. Cells were harvested 38 h later and lysed in 100 μl ice-cold extraction buffer and the protein quantified using the method above. Aliquots of these lysates were mixed with LDS sample buffer (Life Technologies Ltd, cat. number NP0008) and 10X Bolt™ Sample Reducing Agent (Life Technologies Ltd, cat. number B0009) and stored at −20°C, so that the same lysates could be used on every blot.

#### Western blot procedure

A total of 20 µg tissue protein samples or 5 μg cell lysate for controls plus NuPAGE LDS sample buffer and 10X Bolt™ Sample Reducing Agent were loaded onto NuPAGE 4–12% gradient Bis-Tris, 1.0 mm, 15-well mini protein gels (Life Technologies Ltd, cat. no. NP323BOX). SeeBlue Plus2 protein standard (Life Technologies, cat. no. LC5925) was used for estimation of protein molecular weight. Electrophoresis was carried out at 120 V until the dye front reached the bottom of the gel. Protein was transferred to nitrocellulose using NuPAGE transfer buffer (Life Technologies Ltd, cat. no. NP00061) at 30 V for 2 h.

#### Protein detection antibody staining

Nitrocellulose was dried overnight at room temperature and was rehydrated by incubation in PBS for 5 min with gentle rocking and was then rinsed with dH_2_O. It was stained with Revert 700 total protein stain (LI-COR Biosciences UK Ltd, cat. no. 926-11011) for 5 min at RT with gentle rocking, followed by washing with wash solution (60 ml methanol, 13.4 ml acetic acid, 126.6 ml dH_2_O) for 2 × 30 s at RT. Finally, the membrane was rinsed 2× with dH_2_O. Stained membrane was imaged using a ChemiDoc imaging system (Bio-Rad Laboratories) using the StarBright B700 settings.

#### Antibody staining

Nitrocellulose was incubated in blocking buffer (PBS containing 3% powdered skimmed milk) for 30 min at RT and then incubated with primary mouse anti-DOK7 antibody (Santa Cruz, cat. no. sc-390856)^19^ diluted 1:250 in blocking buffer for 1 h at RT with gentle agitation. Membrane was washed 3 × 5 min with 50 ml PBS and then incubated with secondary HRP-conjugated goat anti-mouse antibody (Life Technologies Ltd, cat. no. 31430) diluted 1:100 in blocking buffer for 1 h at RT with gentle agitation. Membrane was washed 3 × 5 min with 50 ml PBS, and HRP activity was detected using ECL™ Start Western Blotting Detection Reagent according to the manufacturer’s instructions (Merck Life Science UK Limited, cat. no. GERPN3243). Stained membrane was imaged using a ChemiDoc imaging system (Bio-Rad Laboratories) using the chemiluminescent settings.

Densitometry of the above staining was carried out using Image Lab 6.1. DOK7 staining was normalized to the total protein stain for each lane, and these values were then normalized with the positive control for that particular blot. This then enabled a semiquantitative comparison of DOK7 levels between different blots.

### Statistical analysis

GraphPad Prism was used for statistical analysis. All error bars shown in figures are standard errors of the mean.

## Results

### Study plan

**Figure 1:**
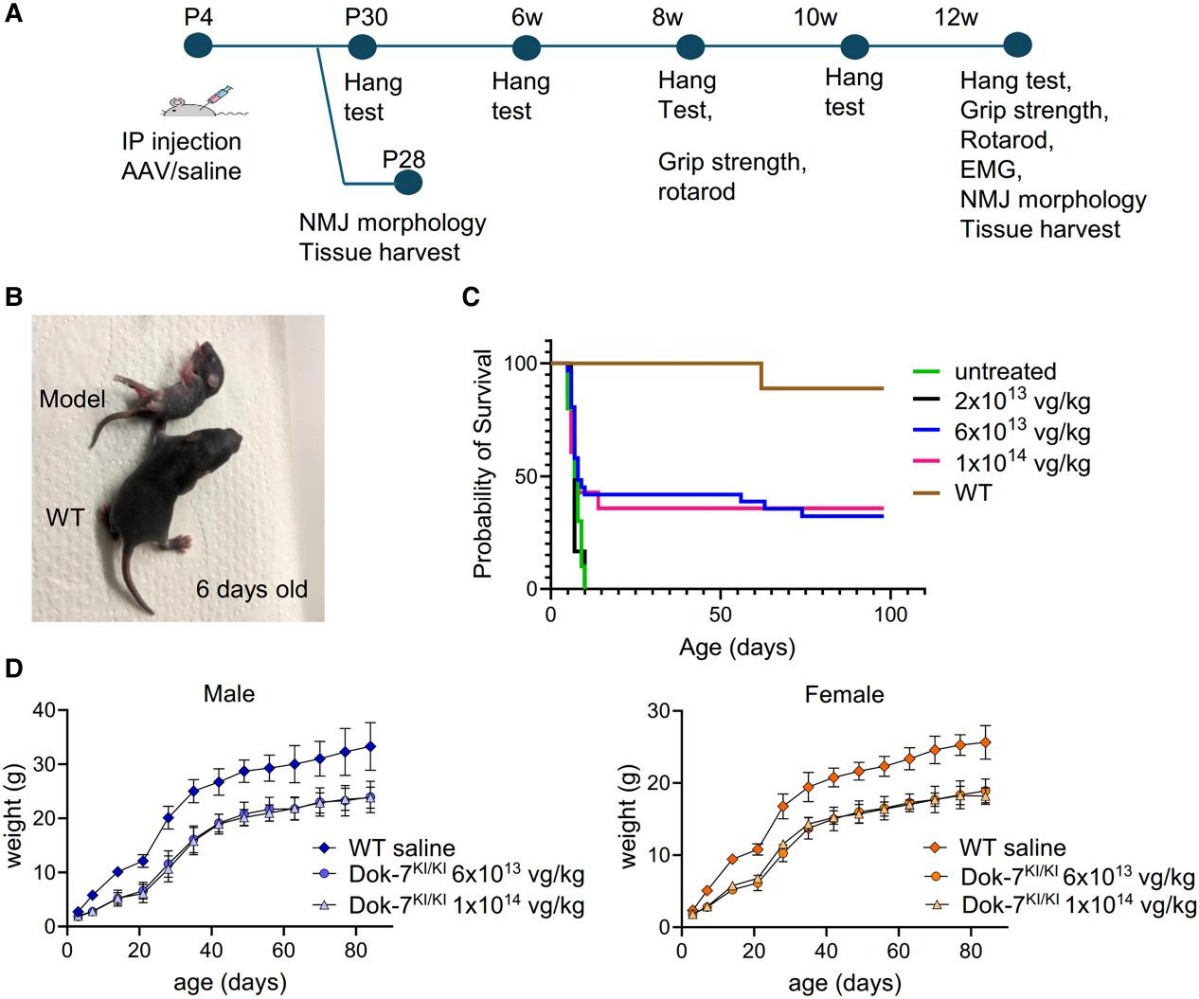
**Timeline, survival and growth of AMP-101-treated Dok-7^KI/KI^ mice.** (**A**) Timeline outlining the study plan, indicating when behavioural tests were carried out. (**B**) Photograph showing the size difference of an untreated Dok-7^KI/KI^ mouse and a WT littermate at 6 days of age. (**C**) Kaplan–Meier survival curve of both genders of Dok-7^KI/KI^ either untreated or treated with various doses of AMP-101 as indicated. Survival curve of saline-injected WT littermates is also shown. (**D**) Male and female growth curves of Dok-7^KI/KI^ mice treated with 6 × 10^13^ vg/kg or 1 × 10^14^ vg/kg and saline-injected WT littermates up to 3 months of age (*n* = 4–5 mice/group). Error bars show standard deviation.

In this dose escalation trial, we administered three different doses of AMP-101 intraperitoneally into 4-day-old Dok-7^KI/KI^ model mice: 2 × 10^13^ vg/kg, 6 × 10^13^ vg/kg and 1 × 10^14^ vg/kg. As controls, a group of Dok-7^KI/KI^ model mice were left uninjected, and WT littermates were injected with saline. Table 1 shows the number of mice and group designation. The number per group was based on our previous experience with this model. The virus was diluted in sterile 0.9% w/v saline by a third party so that the experimenter was blinded to the dose until after data collection and analysis. Mice were culled at either 1 month or 3 months of age. Mouse weight and survival was monitored. For the 3-month groups, muscle fatiguability was monitored every two weeks from 30 days of age using an inverted screen hang test, which is a well-established measure of strength endurance and fatigable weakness. Strength was also assessed by rotarod and grip strength tests at 2 and 3 months of age. EMG was performed at 3 months of age. DOK7 expression in tibialis anterior (TA), diaphragm and triceps brachii was analysed by western blotting for the 3-month-old mice, and the morphology of NMJs in diaphragm was analysed in both the 1- and 3-month groups. The primary efficacy measure was survival and the secondary outcome measure strength as measured by the inverted screen hang test. Figure 1A shows a schematic diagram of the timeline.

**Table 1:** Group designation and number of mice used in the study

Group	Test article	Amp-101 dose vg/kg	Time points	Number of male and female
1	Dok-7^KI/KI^	0	Survival end-point	5 males/5 females
2	WT age matched	Vehicle^a^	1 month, 3 months	4 males/4 females for each time point
3	Dok-7^KI/KI^	6 x 10^13^	3 months	5 males/5 females
4	Dok-7^KI/KI^	2 x 10^13^	3 months	5 males/5 females
5	Dok-7^KI/KI^	1 x 10^14^	3 months	5 males/5 females
6	Dok-7^KI/KI^	6 x 10^13^	1 month	5 males/5 females
7	Dok-7^KI/KI^	2 x 10^13^	1 month	5 males/5 females
8	Dok-7^KI/KI^	1 x 10^14^	1 month	5 males/5 females

^a^Vehicle = sterile 0.9% w/v sodium chloride.

### AMP-101 treatment improved survival and weight gain at higher doses, but not at the lowest dose

The Dok-7^KI/KI^ mouse model used in this study has the common human mutation c.1124_1127dupTGCC knocked in. It has a very severe phenotype, fails to gain weight and dies within a few days of birth (Supplementary Fig. 1). Figure 1B illustrates a comparison between an untreated 6-day-old model mouse and a WT littermate. The lowest dose of 2 × 10^13^ vg/kg did not rescue the severe phenotype of this model in the first six mice injected, and so this group was discontinued for ethical reasons (Fig. 1C, Supplementary mouse weights spreadsheet). For the other two doses, ∼32% of mice treated with 6 × 10^13^ vg/kg and 35% of mice treated with 1 × 10^14^ vg/kg survived beyond 10 days. Of those mice that survived for longer than 10 days, three male mice treated with 6 × 10^13^ vg/kg, one male mouse treated with 1 × 10^14^ vg/kg and one WT male mouse reached the humane end-point before the end of the trial (see Supplementary Table 1 for all causes of premature deaths). Due to the severity of the model, the humane end-point in our ethics protocol was set at: up to P14: no weight gain detected within 2 days, or any weight loss; and after P14: >15% weight loss compared to their previous recorded maximum (Fig. 1C). In the 3-month groups (groups 2, 3 and 5), the surviving AAV-treated model mice gained weight at a similar rate to WT littermates after weaning, but were ∼25% (female) or 28% (male) smaller (Fig. 1D).

### AMP-101-treated Dok-7^KI/KI^ model mice were at least as strong as WT mice for the duration of the study

Three different tests were carried out to assess the muscle strength of the AAV-treated model mice, and male and female mice were analysed separately.

**Figure 2:**
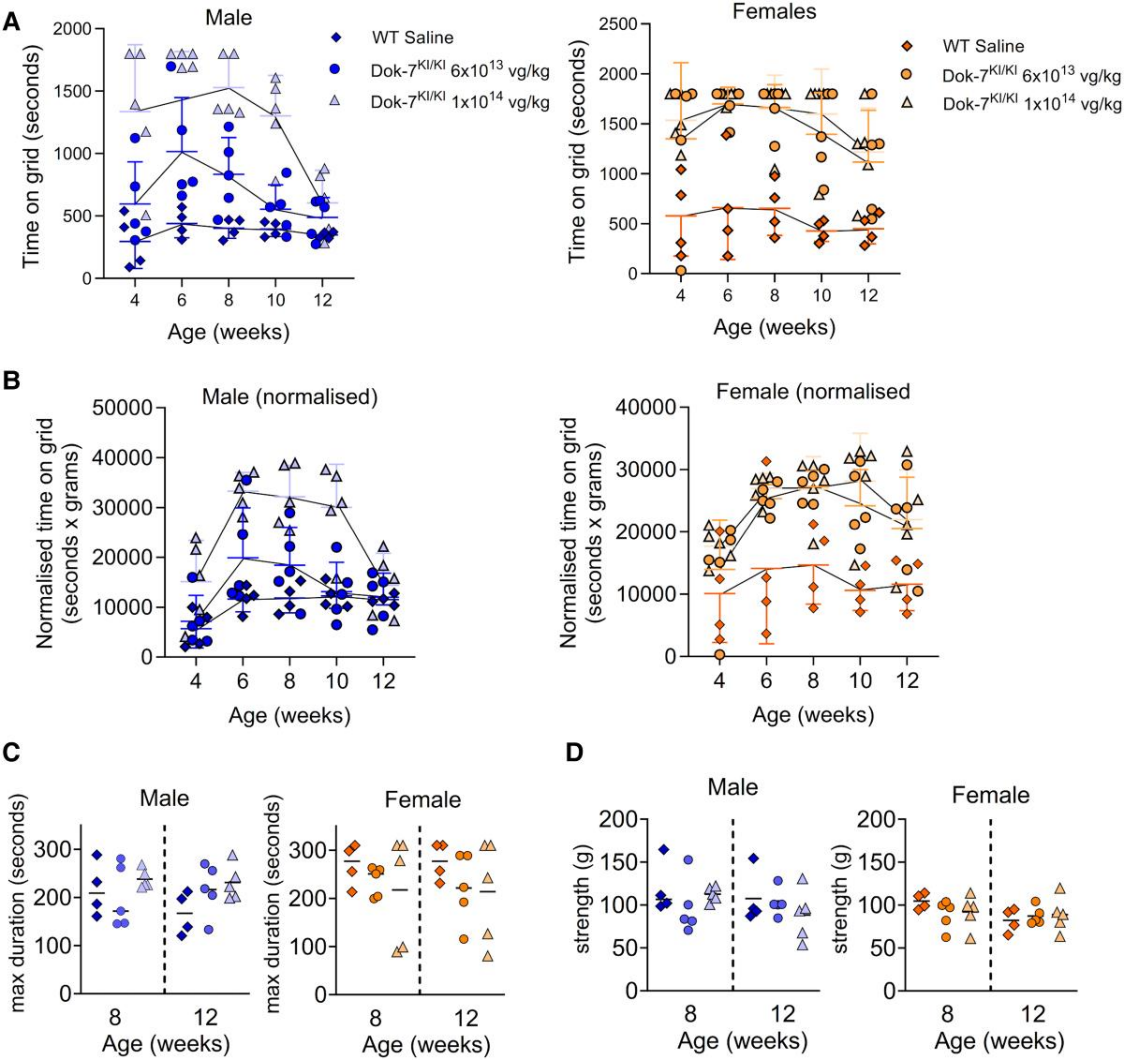
**Strength tests show that AMP-101-treated Dok-7^KI/KI^ mice are as strong or stronger than WT littermates.** (**A**) Inverted screen hang test. Mice were held upside down on a grid and the time to fall was measured. For each mouse at each time point, three consecutive tests were carried out and the cumulative time is shown for each point. (**B**) Normalized inverted screen hang test. To take into account the fact that lighter mice are able to hang onto the screen for longer, the time on grid was normalized by multiplying the time on the screen by the mouse weight. (**C**) Rotarod test. The rotarod speed gradually accelerated from 5 to 40 rpm over a 5 min period. Each test was carried out three times for each time point, and the maximum duration is shown. (**D**) Grip strength test. Mice were gripped by the tail and were allowed to hold onto the grid of the apparatus with their front paws. They were pulled away horizontally and the force generated by the mouse was measured. Each test was carried out five times for each time point, and the maximum value is shown. Each point represents data from an individual mouse at the time point specified (*n* = 4–5 mice). Error bars for **A** and **B** show standard deviation, and lines on **C** and **D** show the mean values.

On fortnightly inverted screen hang tests, models treated with 6 × 10^13^ vg/kg or 1 × 10^14^ vg/kg AMP-101 could hang on for at least as long as WT mice at all time points throughout the trial, with some gender differences (Fig. 2A). Overall, two-way ANOVA tests both doses made model mice significantly stronger than WT mice in both genders (see Supplementary hang test statistical analyses for specific statistical tests conducted and *P*-values). Male models treated with the 1 × 10^14^ vg/kg dose were significantly stronger than those treated with the 6 × 10^13^ vg/kg dose, whereas there was no overall difference in strength between female models treated with the different doses. Muscle strength in treated models appeared to peak at Week 6, after which there appeared a gradual decrease in strength. Sidak’s multiple comparisons showed that decrease was not statistically significant except in male models treated with 1 × 10^14^ vg/kg (*P* = 0.0069). Further comparisons of WT with models treated with either dose at the individual time points showed that there was no significant difference between male WT and models treated with 6 × 10^13^ vg/kg at any time point. However, those models treated with the highest dose of 1 × 10^14^ vg/kg were significantly stronger than WT littermates at all time points except Week 12, and significantly stronger than those treated with 6 × 10^13^ vg/kg at Weeks 8 and 10. In contrast, female models treated with both doses were significantly stronger then WT littermates at Weeks 8 and 10, while those treated with the higher dose were also stronger at 4 weeks. There was no significant difference between the two doses at any time point in female models.

We observed an inverse correlation between weight and hang time in WT mice (Supplementary Fig. 2). As the treated model mice were lighter than WT mice, this might have contributed to their performance on the inverted hang test. To take this into account, we normalized the data by multiplying the hang time by mouse weight (Fig. 2B). Even with this normalization factor, the model mice were still as strong as, or stronger than WT littermates, and the gender differences noted above were largely still observed. The main difference observed when comparing the normalized data with the raw data was that males treated with 6 × 10^13^ vg/kg AMP-101 were not significantly different from WT overall (Supplementary hang test statistical analyses).

For rotarod and grip strength tests, there was no significant difference between treated model mice and WT littermates for either dose at either age and for either gender (ordinary one-way ANOVA, Tukey’s multiple comparisons). In addition, within each treatment group, the strength did not significantly change between 8 and 12 weeks of age (Fig. 2C and D shows data from rotarod and grip tests, respectively).

### AMP-101-treated Dok-7^KI/KI^ model mice show decrement of CMAP on repetitive nerve stimulation

A hallmark of myasthenia is impaired neuromuscular signalling, which can be detected by EMG. This involves repetitively stimulating a motor nerve (in this case the phrenic nerve) and measuring the CMAP after each stimulation. A decrease, or decrement, in successive CMAP amplitudes indicates defective neuromuscular signalling.

**Figure 3:**
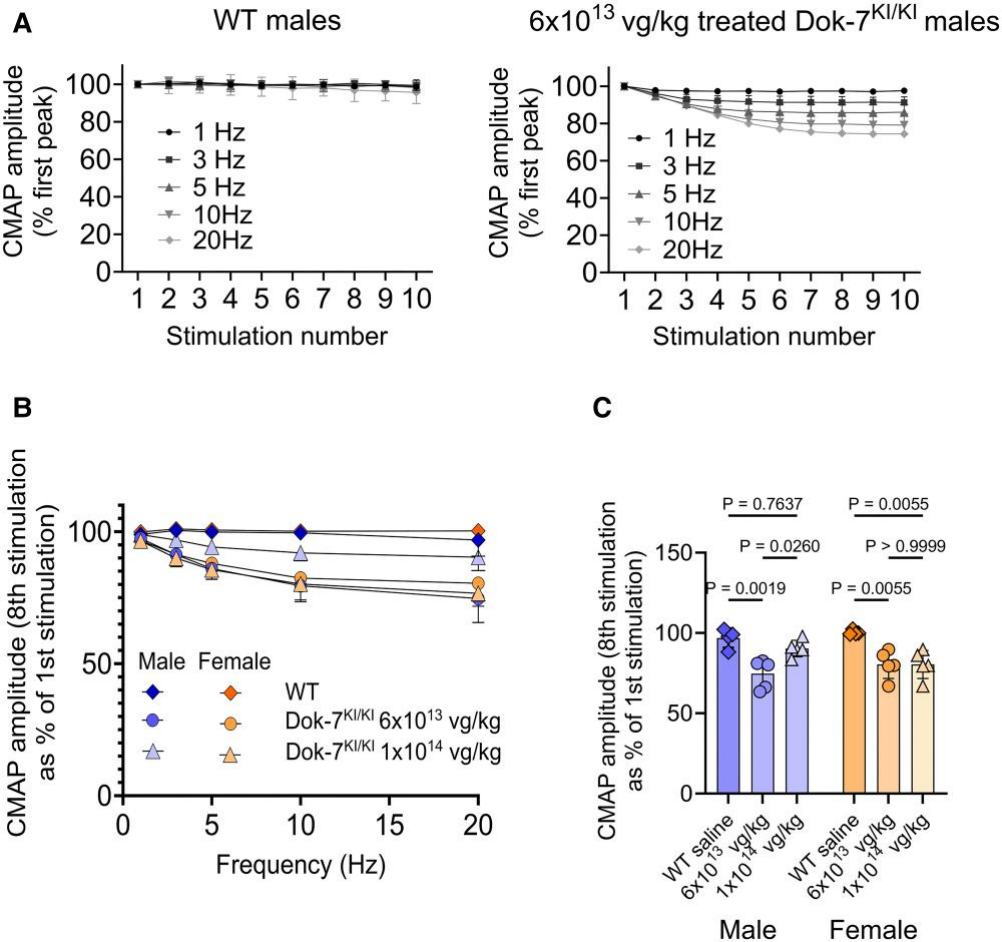
**AMP-101-treated Dok-7^KI/KI^ mice show decrement of compound muscle action potential (CMAP) on repetitive nerve stimulation.** (**A**) Example traces from a WT mouse and an AMP-101-treated model mouse showing the CMAP amplitude as a percentage of the value of the first peak at each stimulus in a train of 10 stimuli at various frequencies as shown. (**B**) CMAP amplitude of the eighth stimulus as a percentage of the first stimulus for each stimulation frequency is shown. The treated model mice show decrement, although the male models treated with 1 × 10^14^ vg/kg do not show significant decrement at 20 Hz compared with WT littermates (**C**). *P*-values were obtained using a one-way ANOVA with Tukey’s multiple comparison test. Error bars show standard deviation, *n* = 3 technical repeats in **A**, *n* = 4–5 mice in **B** and **C**. Bars in **C** represent the mean.

Unfortunately, due to the severity and early lethality of Dok-7^KI/KI^ mice, no EMG data could be collected before treatment, nor on untreated mice. To test whether the AMP-101-treated Dok-7^KI/KI^ mice had impaired neuromuscular signalling, we carried out EMG on 3-month-old mice. The sciatic nerve was given a train of 10 repetitive stimuli at different frequencies, and the CMAP amplitude was measured at each stimulus. Each successive CMAP amplitude was then calculated as a per cent of the first stimulus. Examples of these data for male WT mice and male Dok-7^KI/KI^ mice treated with 6 × 10^13^ vg/kg are shown in Fig. 3A, and Supplementary EMG data.

Even though the treated model mice were at least as strong as their WT littermates, there was nonetheless evidence of decrement of up to 25% at 20 Hz. WT littermates did not exhibit decrement (Fig. 3B, Supplementary Fig. 3). It is of note that, out of the treated models, the males treated with the highest dose of 1 × 10^14^ vg/kg showed the least decrement (only 10% at 20 Hz, Fig. 3C, Supplementary Fig. 3) and this was not significantly different from WT (*P* = 0.7637; one-way ANOVA, Tukey’s multiple comparison test).

### Enlarged NMJs are observed following treatment with AMP-101

**Figure 4:**
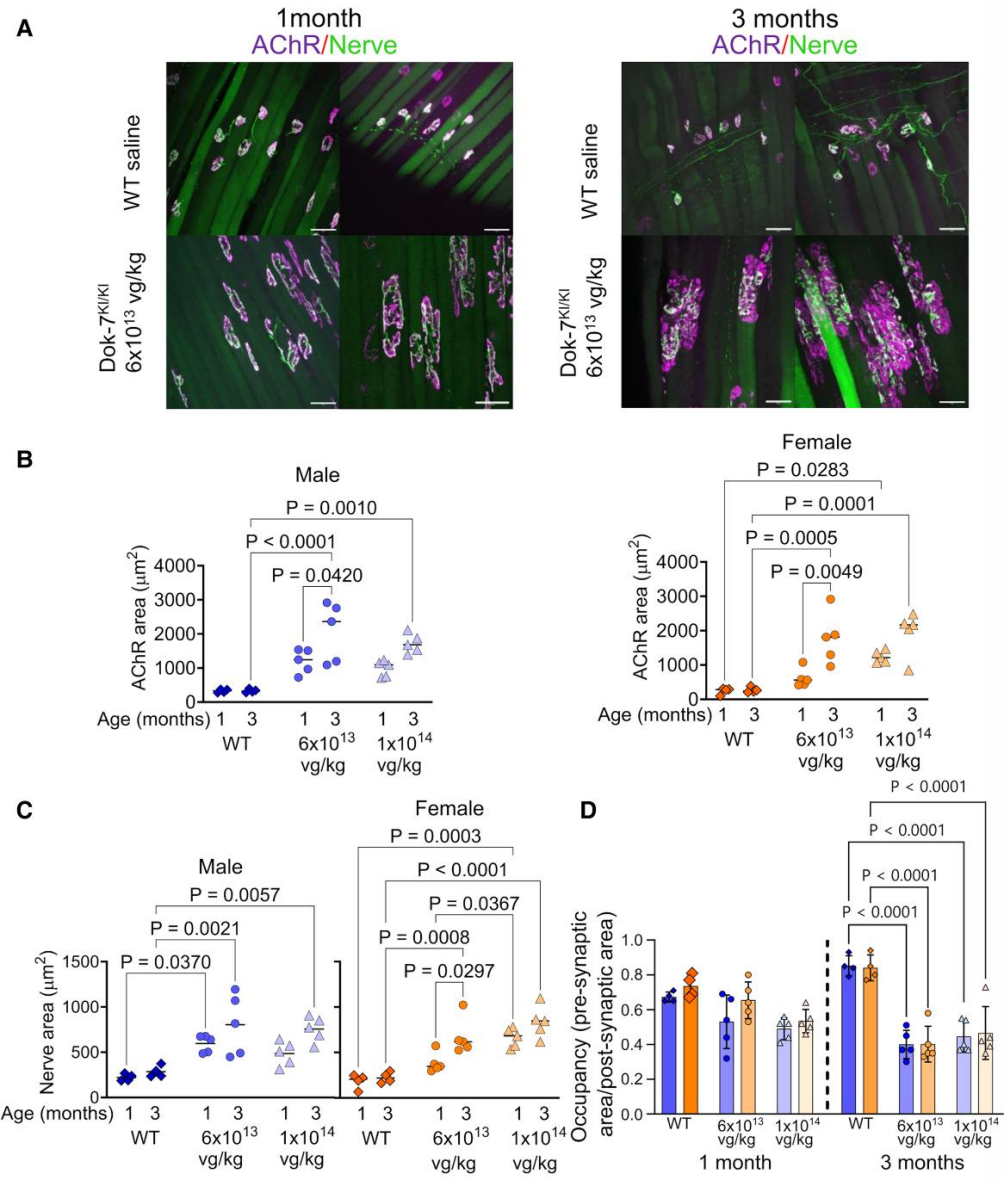
**AMP-101 generates enlarged neuromuscular junctions in Dok-7^KI/KI^ model mice.** (**A**) Example maximum projection images generated from confocal z-stacks of diaphragm muscle from WT mice and from Dok-7^KI/KI^ mice treated with 6 × 10^13^ vg/kg of AMP-101. Motor neuron termini are stained with antibodies against neurofilament and synaptophysin ("Nerve"), and post-synaptic nAChRs are stained with a fluorescent α-bungarotoxin ("AChR"). Scale bars indicate 50 µm. (**B** and **C**) NMJs are enlarged in AMP-101-treated model mice 1 month after treatment compared with WT littermates, and continue to enlarge as the mice grow to 3 months of age. The graphs show post-synaptic nAChR area (**B**) and pre-synaptic area of nerve (**C**). Bars indicate mean values. (**D**) At 1 month of age, the ratio of pre- to post-synaptic staining, known as occupancy, is not significantly different between WT and treated models. By 3 months of age, occupancy is significantly lower in treated models compared with WT littermates. *P*-values were obtained using a one-way ANOVA with Tukey’s multiple comparison test. Error bars show standard deviation and bars represent mean values. In **B–D**, each point represents mean of diaphragm NMJ measured in each mouse (∼20), *n* = 4–5.

Previous publications and our own observations have shown that overexpression of DOK7 in mouse skeletal muscles gives rise to enlarged NMJs,^17,18,30,31^ and we wanted to know whether this novel rAAV construct would also give rise to enlarged synapses. Diaphragms collected from mice one or three months after treatment were stained with antibodies against neurofilament and synaptophysin to visualize the pre-synaptic motor neuron terminal, and post-synaptic nAChRs were stained with fluorescent 594-α-BuTx. Enlarged synapses were present in all AMP-101-treated model mice at both one and three months of age (Fig. 4A shows example images, NMJ area measurements and comparisons in Supplementary Table 2). Variation in the size of NMJs on different muscle fibres in the same diaphragm was observed.

Gender-specific differences were examined across various groups. In WT control mice, there was no significant disparity in the size of the NMJ between males and females at either age (Supplementary Table 2, Supplementary Fig. 4). In contrast, gender-specific differences were detected in the treated model mice at 1 month of age. Specifically, in model mice treated with 6 × 10^13^ vg/kg, the NMJs were, on average, larger in males than females, as evidenced by both 594-α-BuTx and nerve staining. However, in mice treated with 1 × 10^14^ vg/kg, females exhibited larger pre-synaptic areas. By 3 months of age, no significant gender differences were detected in either pre- or post-synaptic staining. Given the gender disparity identified at 1 month of age, we further analysed the males and females separately.

In WT mice, the average NMJs’ area stained with 594-α-BuTx was ∼330 μm^2^ in males and 250 μm^2^ in females and no change in size was observed between 1 and 3 months of age. In 1-month-old DOK-7^KI/KI^ mice treated with AMP-101, the NMJs were larger than those of WT littermate controls with the largest being in females treated with the highest dose of 1 × 10^14^ vg/kg (mean α-BuTx area of 1242 μm^2^). NMJs in treated model mice increased in size between 1 and 3 months, although this change was not always statistically significant (Fig. 4B, one-way ANOVA with Tukey’s multiple comparison test). We were interested in whether the dose influenced the size of the NMJs, but the only significant difference between doses was in the nerve staining in the female model mice at 1 month of age, with 1 × 10^14^ vg/kg generating the larger area of 663.4 μm^2^ compared with 384 μm^2^ for 6 × 10^13^ vg/kg (Fig. 4B, *P* = 0.0367; one-way ANOVA with Tukey’s multiple comparison test).

In WT NMJs, there is a good registration between the pre- and post-synaptic staining. This relative localization can be quantified as NMJ occupancy by calculating the ratio of pre- and post-synaptic area. For WT mice, the NMJ occupancy is ∼0.7 at 1 month, increasing to around 0.85 by 3 months (Fig. 4C). In the treated models at 1 month of age, NMJ occupancy is not significantly different to WT mice. However, at 3 months of age, NMJ occupancy in AMP-101-treated models is significantly lower than for WT littermates (∼0.4 for 6 × 10^13^ vg/kg and 0.45 for 1 × 10^14^ vg/kg for both genders). This reflects the fact that at 3 months of age, the post-synaptic area has increased to a larger extent than the pre-synaptic area, and therefore a lower proportion of the post-synaptic density is covered by the motor neuron terminus. This can be observed in Fig. 4A.

### Human DOK7 is expressed in several different muscles

AMP-101 was injected into the peritoneum and so we expected that the diaphragm would be readily accessible to the virus, as borne out by the presence of enlarged synapses in this muscle. However, it was important to assess gene expression in other muscles more distal from the injection site. Therefore, the forelimb triceps brachii and hindlimb TA from the 3-month-old groups were selected for further investigation by western blotting.

**Figure 5:**
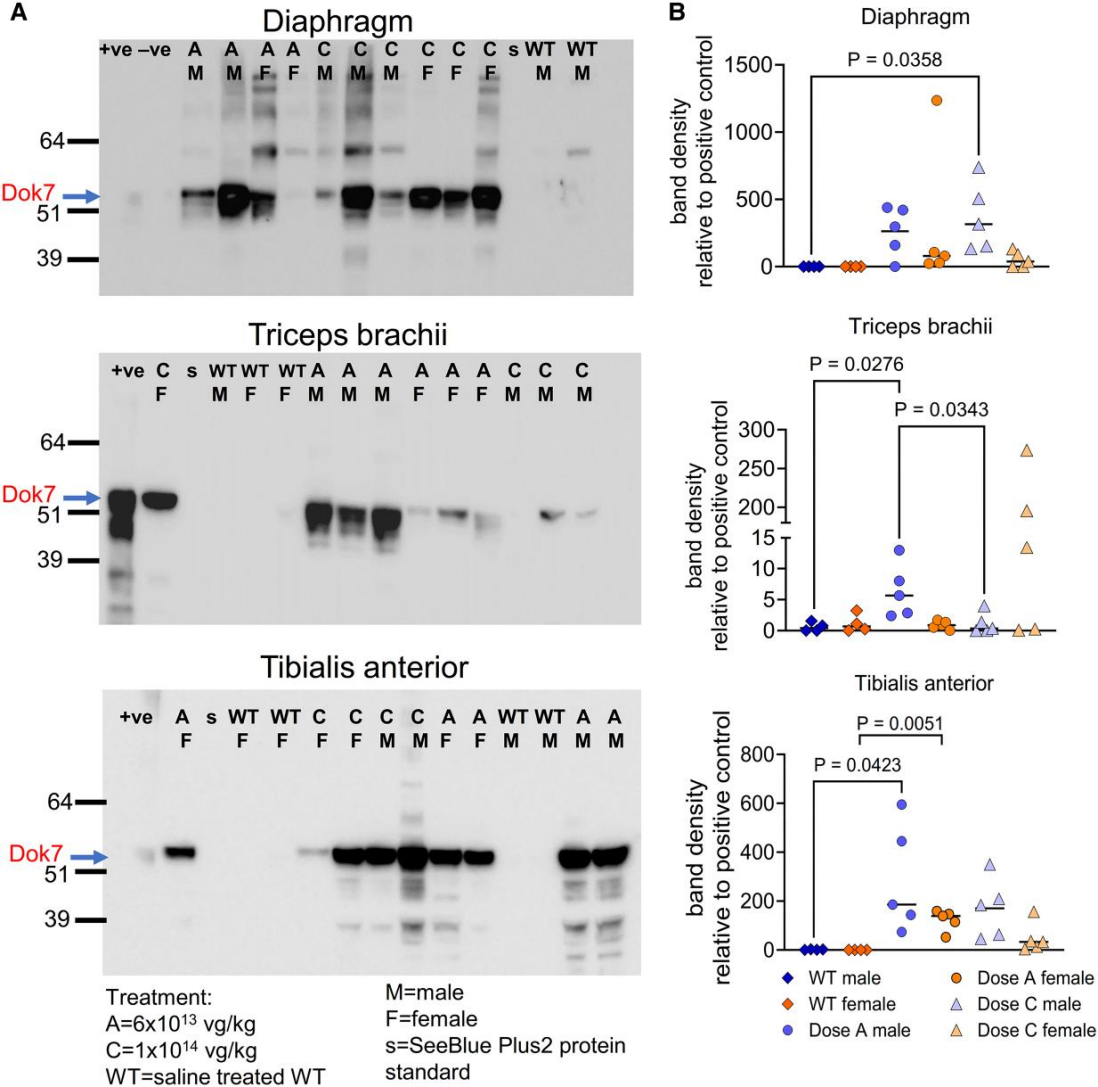
**DOK7 is expressed in diaphragm, triceps brachii and TA in AMP-101-treated Dok-7^KI/KI^ model mice.** (**A**) Examples of western blots showing DOK7 expression. Robust expression is seen in muscles from many of the treated mice. Endogenous levels of DOK7 in WT mice are very low. (**B**) Densitometry was carried out to semi-quantify DOK7 expression. First, the ratio of the band intensity of DOK7 with total protein was calculated, and then this was normalized with a positive control (lysate from HEK292T cells transfected with human DOK7 cDNA). Each point represents a mouse, *n* = 4–5. *P*-values were obtained using a one-way ANOVA with Tukey’s multiple comparison test. Bars show mean values.

DOK7 protein was detected in most of the treated model mice and was virtually undetectable in the WT littermates. However, the levels of expression in treated models varied greatly and this large variation did not seem to correlate with dose. Example blots showing staining of DOK7 are shown in Fig. 5A and Supplementary Fig. 6. To semi-quantify DOK7 protein expression, the DOK7 band intensity was normalized to total protein in each lane (visualized by using Revert700—examples shown in Supplementary Fig. 5) and then normalized with a positive control loaded onto each gel (Fig. 5B). From these data, it is clear that AMP-101 injected into the peritoneum is able to transduce muscles that are located in different parts of the body, namely diaphragm, triceps brachii and TA, and express DOK7.

## Discussion

The aim of this project was to test the efficacy of a novel AAV gene therapy, AMP-101, in a severe mouse model of DOK7-CMS. We have demonstrated that when AMP-101 was injected intraperitoneally, it could transduce different muscles and generate enlarged synapses. Most remarkably it could rescue this extremely weak and fragile mouse, increasing survival and restoring normal strength.

Recombinant AAV virus is a leading delivery vector of gene therapies and has been widely used in over 250 clinical trials (see Shen *et al*.^32^ for meta-analysis). Seven different rAAV gene therapies have reached the market; Roctavian™ (BioMarin) for treatment of haemophilia A and Hemgenix® (uniQure/CSL) for haemophilia B,^33^ Elevidys® (Sarepta Therapeutics) to treat Duchenne muscular dystrophy,^34^ Luxturna™ (Spark Therapeutics Inc) for treatment of retinal dystrophy,^35^ Zolgensma® (Novartis Gene Therapies) for spinal muscular atrophy,^36^ Glybera® (Uniqure) to treat lipoprotein lipase deficiency (although it is no longer marketed)^37^ and Upstaza™ (PTC Therapeutics) as a treatment for aromatic L-amino acid decarboxylase deficiency.^38^ Although these successes highlight their therapeutic benefit, the use of rAAV is linked to some adverse effects, rAAV-mediated hepatoxicity is the most common adverse effect, and dorsal root ganglia toxicity has been observed after CNS-targeted systemic gene transfer in non-human primates.^39,40^ Adverse events linked to severe hepatobiliary syndrome and sepsis have caused the death of four children enrolled in the ASPIRO clinical trial (NCT03199469) aiming to show the safety and efficacy of an AAV8 based gene therapy (AT132) for the treatment of X-linked myotubular myopathy (XLMTM).^41^ The patients that unfortunately died were older and heavier and thus received among the highest total vg (range: 4.8 × 10^15^/7.74 × 10^15^ total vg). Moreover, they had evidence of pre-existing intrahepatic cholestasis that may have played a pathogenic role.^42^

It is therefore preferable to develop rAAV-based therapies with a lower minimum therapeutic dose (MTD). Here, none of the first six animals injected with a dose of 2 × 10^13^ vg/kg survived past two weeks of age, and further injections at this dose were stopped on ethical grounds. Successful treatment was observed in 32% and 35% of the model mice injected with 6 × 10^13^ and 1 × 10^14^ vg/kg, respectively. This is lower than the survival rate of 100% described by Arimura *et al*.^17^ when the DOK-7^KI/KI^ mouse model was injected with an AAV9 vector in which human DOK7 was under control of the CMV promoter. It is not clear why 65% of mice reported here failed to thrive, but it could be a combination of several factors. We used an AVVrh74 vector, and although AAVrh74 is able to transduce striated muscle, the AAV9 serotype might have slightly increased early transduction.^43^ Arimura *et al*. also used a CMV promoter whereas our construct contained a muscle-restricted promoter, which may have different transcription activation kinetics. The most important factor is likely to be the difference in severity of the DOK-7^KI/KI^ model in our lab versus in Prof. Yamanashi’s lab. Although we obtained the model from Prof. Yamanashi, our strain was outbred for one generation with C57BL/6J mice, so the background strain of the mice used here would be slightly different to that used by Arimura *et al*.,^17^ further diluting the original strain they used to generate the model. As Oury *et al*.^44^ showed, the background strain can modify the severity of the DOK-7^KI/KI^ model, with a background of C57BL/6J having the most severe phenotype of the strains they tested, dying a day after birth. Unfortunately, our study was initiated prior to this publication, so it was too late to outbreed our strain. Our untreated DOK-7^KI/KI^ mice survived fewer than 10 days whereas Arimura *et al*. reported survival between 13 and 20 days of age. This meant that there was a shorter window of opportunity for the rAAV to transduce the muscle, express DOK7 and remodel the NMJs to become large enough to rescue the phenotype. We injected the pups at 4 days of age to maximize this window, but this still only gave the treatment < 5 days to take effect. An earlier injection would likely have given the gene therapy more time to take effect, and may have saved more mice. However, it would have required handling of weak pups even earlier, which may have caused more maternal rejections or cannibalism.

Nevertheless, nearly all the animals that did survive past two weeks of age survived for the duration of the study, which is clearly a better outcome than uninjected mice (Supplementary Table 1). Moreover, each of the muscle groups we looked at showed effective levels of transduction and, given the survival and strength of the mice, it is likely that all key muscle groups throughout the animals were effectively transduced. It is important to note that this mouse model exhibits a much more severe phenotype than DOK7-CMS patients, and therefore the survival rate for this model does not directly transfer to the clinical context.

Of the mice that survived more than 2 weeks, three males (two injected with 6 × 10^13^ vg/kg and one injected with 1 × 10^14^ vg/kg) reached the humane end-point for weight loss later in the trial. In addition, one male WT mouse also reached the weight loss humane end-point and had to be culled. We could not ascertain a reason for this weight loss, and there was no visible evidence of liver disease during the necropsy, or dental problems that may have affected the ability to eat. It is possible the deaths may have been unrelated to the transgenic status or the therapy, especially since a WT male also lost weight and reached the humane end-point. Liver toxicity studies on AMP-101 did not detect any adverse effects related to the treatment (Supplementary Data, Supplementary Tables 3–5).

The most important finding from a clinical perspective is that all the mice injected with AMP-101 showed WT levels of strength or better in all the strength tests carried out. The effect of the treatment appeared to peak at 6 weeks in most treatment groups, and appeared to decline in male mice, but this decline was only significant in male models injected with 1 × 10^14^ vg/kg dose. Although male mice injected with 1 × 10^14^ vg/kg were stronger than those injected with 6 × 10^13^ vg/kg overall, the only significant differences in performance on strength tests at individual time points were in Week 8 and Week 10 hang tests. For the female treated models, there was no difference in strength between the two doses at any age. These data indicate that the MTD for AMP-101 would be around 6 × 10^13^ vg/kg, which is lower than the recommended dose for Zolgensma® (1.1 × 10^14^ vg/kg). It should also be noted that we had to inject AMP-101 intraperitoneally because the 4-day-old pups are too small for IV injection, the preferred route of administration in patients. Nonetheless, the relatively low MTD of AMP-101 makes it promising as a potential therapy for DOK7-CMS.

Even though the treated model mice did not show any sign of fatiguability on the inverted screen hang test, EMG analysis indicated that there was a neuromuscular signalling defect in most groups. Decrement indicates a drop-off of muscle fibre recruitment with successive stimuli. As the transduction efficiency of AAVrh74 is not perfect, some fibres may not have been transduced by AMP-101 and might have no NMJs, or some NMJs might be too small to repeatedly generate action potentials. This is supported by the fact that there was a wide variation in NMJ sizes in all the treated model mice. The fact that the mice are strong suggests that even if this is the case, sufficient muscle fibres are transduced to restore and maintain normal strength. The CMAP amplitude in male mice treated with the highest dose of 1 × 10^14^ vg/kg did not decrease significantly below that of WT littermates, indicating that this dose successfully ameliorated decrement in male mice, but not in females.

The AAV genome does not integrates into the cell genome, but remains episomal in the nucleus. Thus, in rapidly dividing cells, the AAV DNA will become diluted and less effective with each cell division. AAV gene therapy is therefore likely to be more effective in tissue that contain slowly or non-dividing cells, such as skeletal muscle. However, the effectiveness may reduce if the AAV is administered in infancy/childhood (as would be preferred for treatment of CMS), because the muscle will grow and increase in mass, reducing the viral genome to host genome ratio. This may explain why the strength of our treated animals reduced from 6 to 12 weeks, and more so in males than females as males grew bigger than females (weighing 13 times more on average than at time of injection, compared to 10 times in females). However, the size of NMJs in treated models at 1 month and 3 months of age did not show any evidence of such a dilution effect. In fact, the NMJs increased during this period, demonstrating AMP-101 continued to induce enough DOK7 expression to maintain NMJ function. Another factor to consider is an observed reduction in registration between the nerves and post-synaptic density of the NMJ between 1 month and 3 months. This reduced registry is likely caused by the large expansion of the post-synaptic area, and although the pre-synaptic area also increased, it may have struggled to keep up with this.

These data show that AMP-101 is clearly on a different efficacious level to the current preferred clinical treatment, salbutamol, which only extends the lifespan of this mouse model by a maximum of 3 days.^45^ It is more difficult to compare its efficacy to another new treatment that effectively rescued a severe mouse model of DOK7-CMS—MuSK agonist antibodies pioneered by Prof. Steve Burden’s group.^44^ Despite having the same DOK7 c.1124-1127dupTGCC variant knocked in, Prof. Burden’s group used a mixed C57BL/6-CBA background to test MuSK agonist antibodies, which had a longer survival time than our C57BL6J mice. The MuSK agonist antibodies rescued the survival of all the model animals, and improved synapse size, though not to WT levels. Our studies also present motor performances slightly differently, with the Oury *et al*. study dividing motor performance by the weight of the animals, which are significantly less for model animals than WT, whereas hang times were multiplied by the weight in this study due to the negative correlation we found between weight and hang test performance (Supplementary Fig. 2). However, one may notice from the Supplementary Video that MuSK agonist-injected animals had splayed hind limbs, which did not tuck under the body to move in a normal upright gait, whereas AMP-101-treated mice had normal upright gait and movement (Supplementary Video).

In summary, we have demonstrated that AMP-101, a novel rAAV expressing human DOK7, is an effective gene therapy in a mouse model for DOK7-CMS when administered at doses comparable with or lower than current rAAV therapies that are already in the clinic. We also show that the treatment applied at a very young age alleviated the disease phenotype even after the animals grew to adulthood, producing WT levels of strength. This compares favourably to the best current treatment for patients, salbutamol, and the other exciting new treatment for DOK7-CMS—MuSK agonist giving hope that a one-off treatment will bear long-lasting effects.

## Supplementary Material

fcaf046_Supplementary_Data

## Data Availability

The authors confirm that the data supporting the findings of this study are available within the article and its Supplementary material. Raw data are presented in supplementary spreadsheets and are available upon reasonable request from the corresponding author.
