# Supplementary material for: Dose escalation pre-clinical trial of novel DOK7-AAV in mouse model of DOK7 congenital myasthenia
Source: Brain Commun. 2025 Jan 30;7(1):fcaf046. doi: 10.1093/braincomms/fcaf046 (PMC11814498; doi:10.1093/braincomms/fcaf046)
Supplement: fcaf046_Supplementary_Data [file fcaf046_supplementary_data.zip › Supplementary Material.pdf]

Supplementary Material

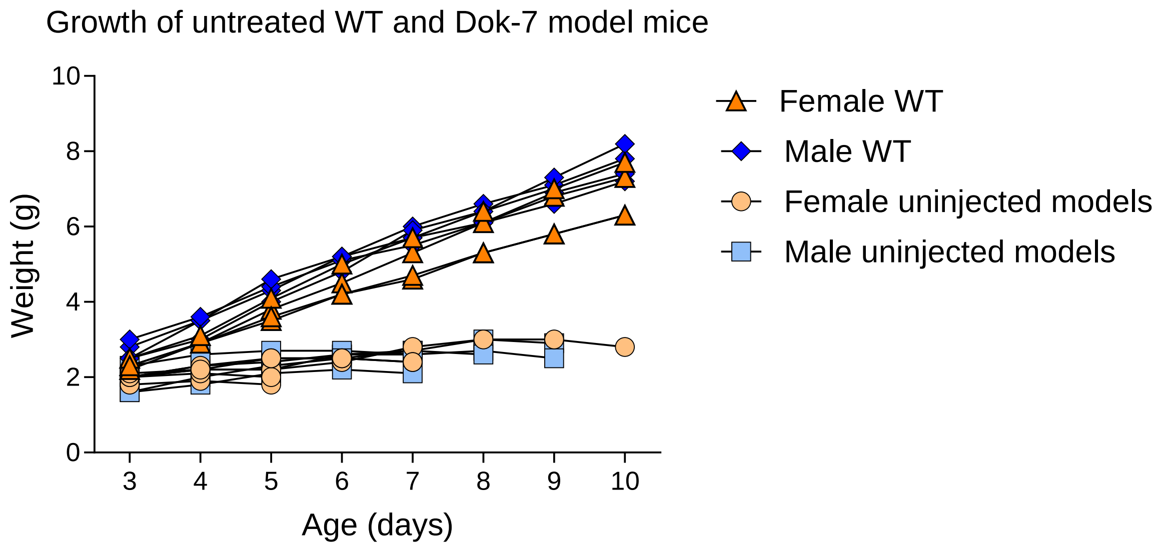

Supplementary Figure 1. Untreated Dok-7$^{KI/KI}$ pups gain very little weight compared with WT littermates.

Growth curve of male and female WT and untreated model mice from P3 to P10. The model mice fail to thrive and hardly grow at all. Each point represents a weight measurement for an individual mouse.

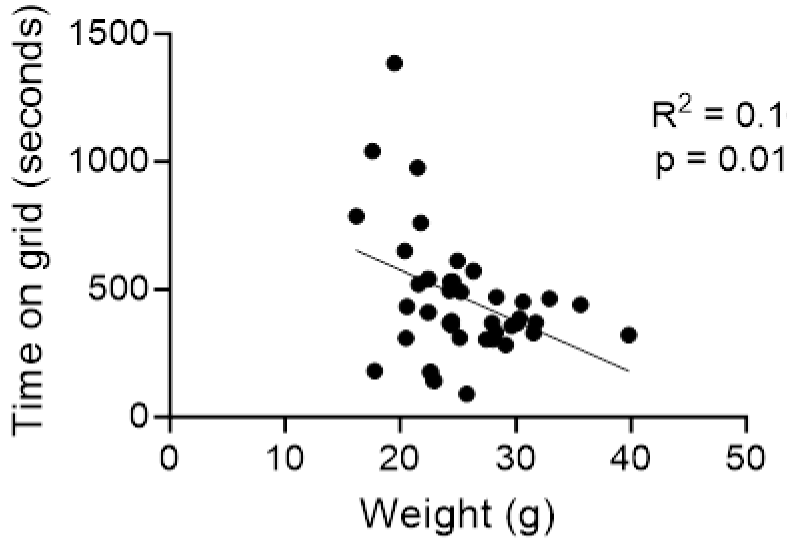

Supplementary Figure 2. Length of time WT mice can hang onto an inverted screen is inversely proportional to weight.

This correlation graph shows that heavier WT mice hold onto an inverted screen for less time than lighter mice. Each point represents cumulative hang time from three sequential attempts from a mouse.

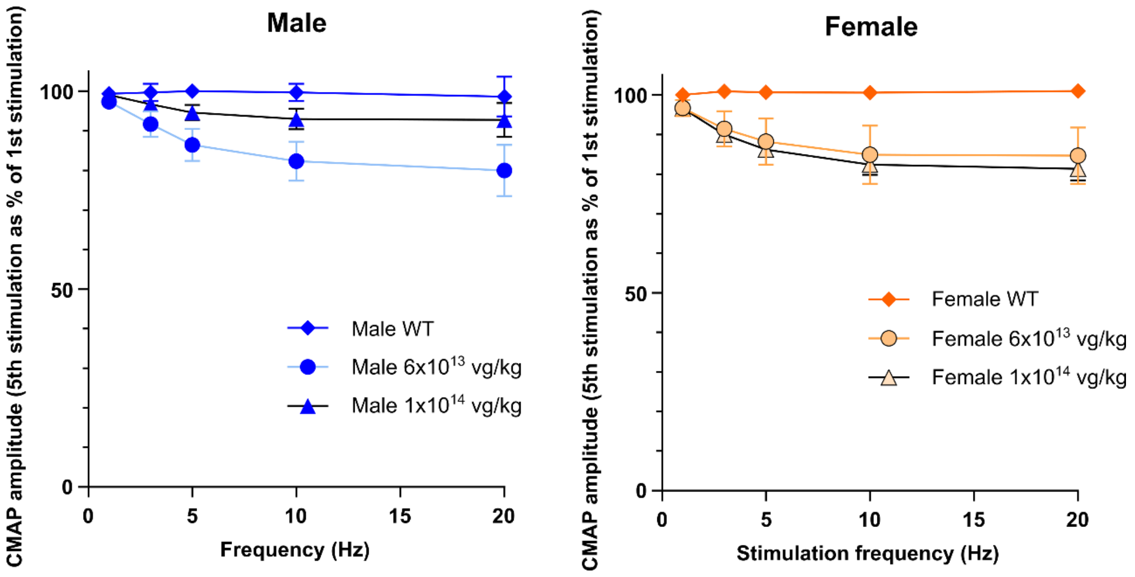

Supplementary Figure 3. Decrement in CMAP when looking at the 5th stimulation as a proportion of the 1st stimulation. In clinical EMG, decrement is often determined by looking at the CMAP amplitude of the 5th stimulation as a proportion of the 1st stimulation, where >10% decrement is considered significant. By this criteria, significant decrement was observed in AMP-101 injected mice, except for 1x10^{14} vg/kg injected male mice. Each point represent the mean of each group, n = 4-5. Error bars represent standard deviation.

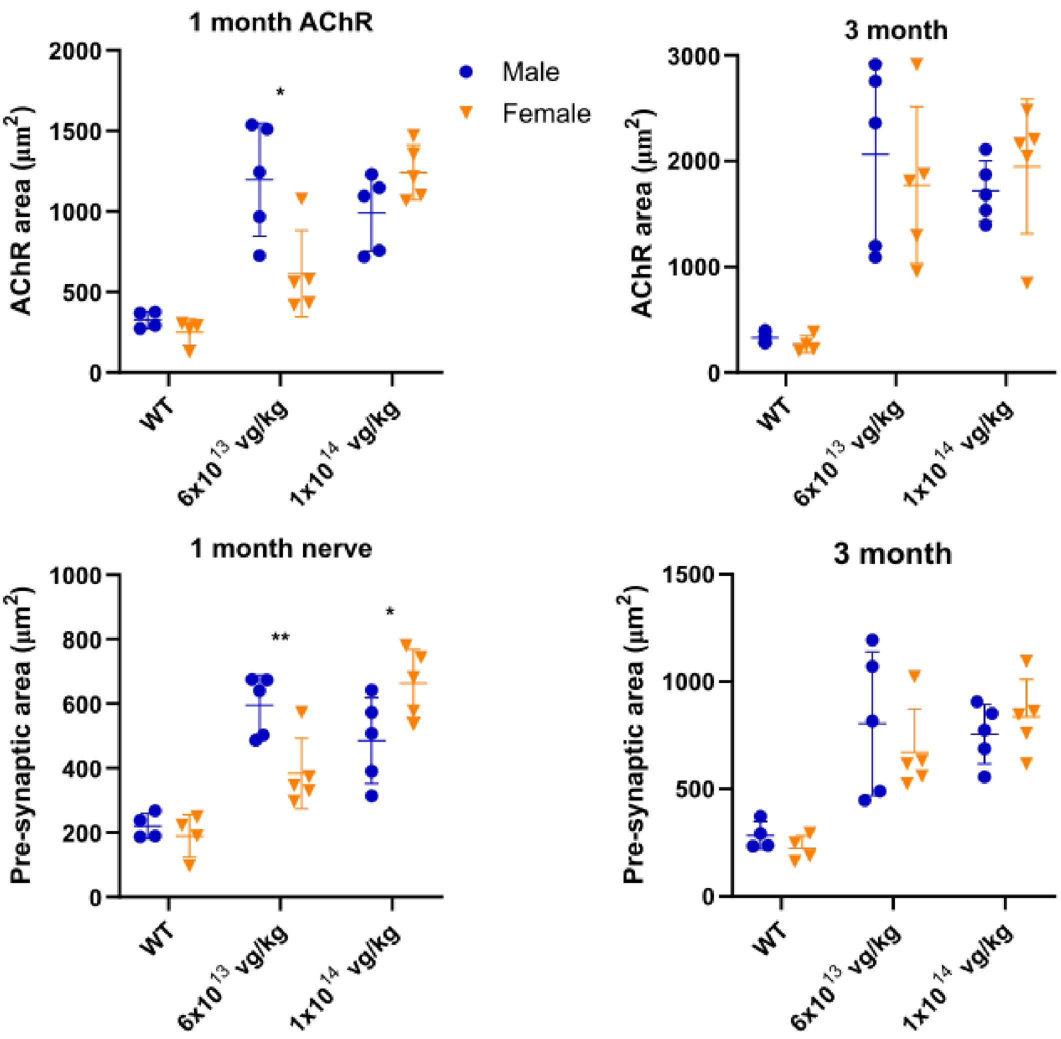

Supplementary Figure 4. Gender differences in the size of pre-and post-synaptic areas.

At 1 month of age (but not 3 months) AMP-101 treated male and female model mice have significantly different mean AChR areas (* $p = 0.0138$ for 6x10e^{13}vg/kg) and nerve areas (** $p = 0.0069$ for 6x10e^{13} vg/kg, * $p = 0.0204$ for 1 x 10^{14} vg/kg). There is no significant difference between WT male and WT female mice at either age. Two-way ANOVA with Sidak's multiple comparison test. Each point represents mean NMJ measurement for each mouse. Error bars show standard deviation.

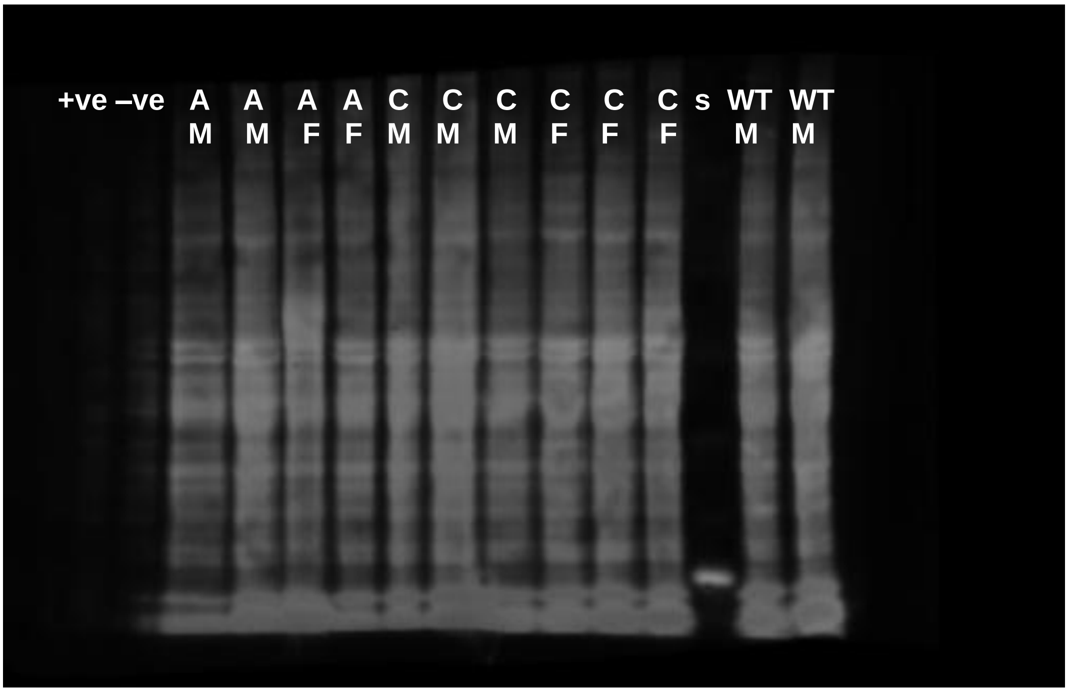

Supplementary Figure 5. Example of protein stain on nitrocellulose using Revert 700 protein stain.

This shows the Revert 700 protein stain of some of the extracts from mouse diaphragm. It is the same nitrocellulose blot that is shown in Figure 5. A=$6x10^{13}$ vg/kg; C=$1x10^{14}$ vg/kg; WT=saline treated WT; M=Male; F=Female; s=SeeBlue Plus2 protein standard; +ve = lysate from HEK293T cells transfected with human DOK7 cDNA; -ve=lysate from mock transfected HEK293T cells.

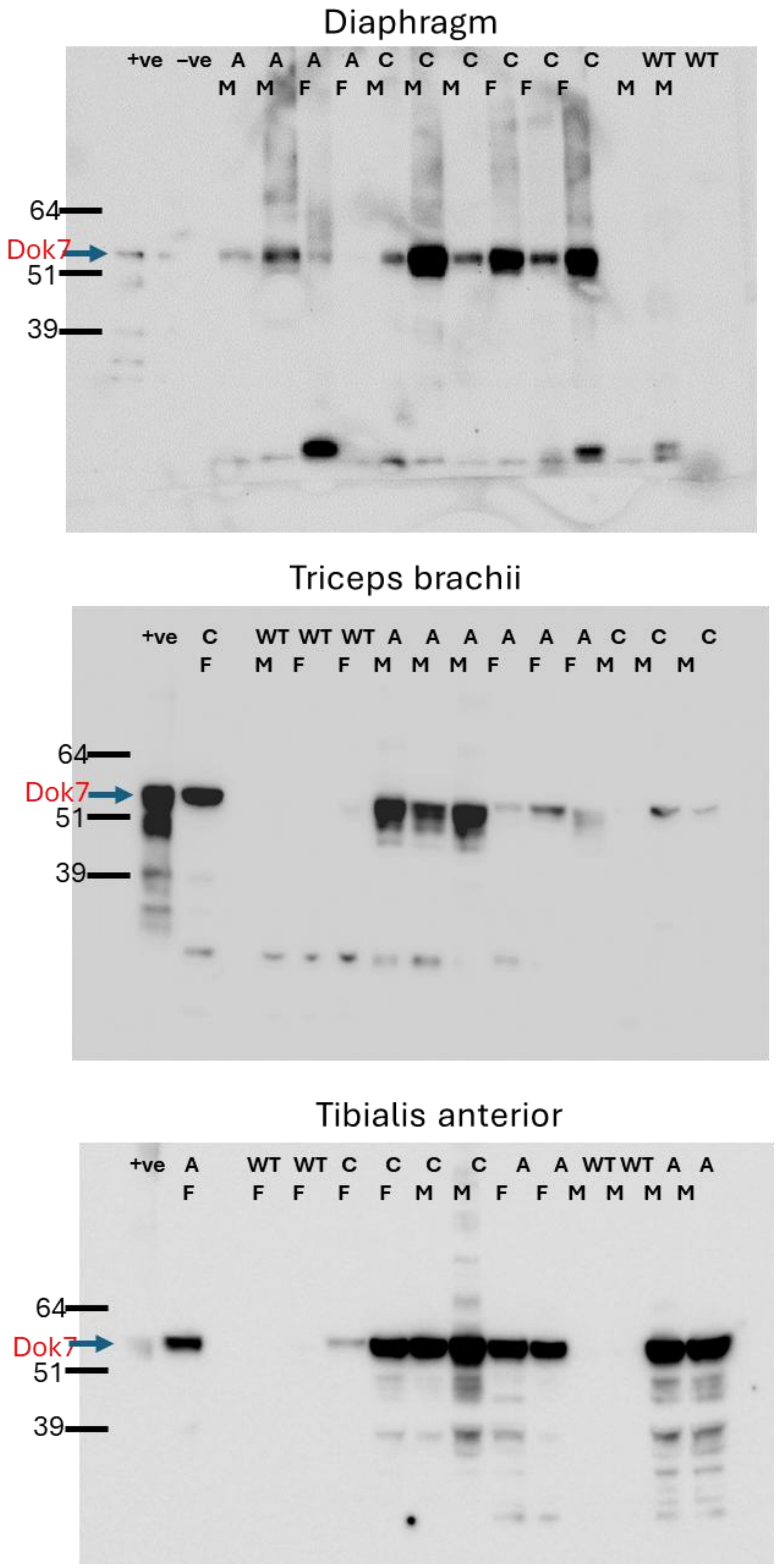

Supplementary Figure 6. Uncropped images of western blots presented in Figure 5. A=6x10^{13} vg/kg; C=1x10^{14} vg/kg; WT=saline treated WT; M=Male; F=Female; s=SeeBlue Plus2 protein standard; +ve = lysate from HEK293T cells transfected with human DOK7 cDNA; -ve=lysate from mock transfected HEK293T cells.

Supplementary Table 1. Causes of premature deaths

gender	dose	age at death/days	cause of death
Male	WT	63	culled - reached human endpoint (15% less weight than recorded maximum)
Male	$2x10^{13}$ vg/kg	6	culled - reached human endpoint (lost weight before P14)
Male	$2x10^{13}$ vg/kg	7	culled - reached human endpoint (no weight gain within 48 hours)
Male	$2x10^{13}$ vg/kg	6	culled - reached human endpoint (lost weight before P14)
Male	$6x10^{13}$ vg/kg	49	culled - reached human endpoint (15% less weight than recorded maximum)
Male	$6x10^{13}$ vg/kg	70	culled - reached human endpoint (15% less weight than recorded maximum)
Male	$6x10^{13}$ vg/kg	63	culled - reached human endpoint (15% less weight than recorded maximum)
Male	$6x10^{13}$ vg/kg	6	went missing
Male	$6x10^{13}$ vg/kg	8	culled - reached human endpoint (lost weight before P14)
Male	$1x10^{14}$ vg/kg	8	culled - reached human endpoint (no weight gain after 48 hours)
Male	$1x10^{14}$ vg/kg	8	culled - reached human endpoint (no weight gain after 48 hours)
Male	$1x10^{14}$ vg/kg	7	found dead
Male	$1x10^{14}$ vg/kg	5	culled - reached human endpoint (no weight gain after 48 hours)
Male	$1x10^{14}$ vg/kg	9	found dead
Male	$1x10^{14}$ vg/kg	8	culled - reached human endpoint (no weight gain after 48 hours)
Male	$1x10^{14}$ vg/kg	5	went missing
Male	$1x10^{14}$ vg/kg	13	culled - reached human endpoint (lost weight before P14)
Female	$2x10^{13}$ vg/kg	7	culled - reached human endpoint (no weight gain after 48 hours)
Female	$2x10^{13}$ vg/kg	11	culled - reached human endpoint (lost weight before P14)
Female	$2x10^{13}$ vg/kg	7	culled - reached human endpoint (lost weight before P14)
Female	$6x10^{13}$ vg/kg	4	found dead
Female	$6x10^{13}$ vg/kg	8	went missing
Female	$6x10^{13}$ vg/kg	6	culled - reached human endpoint (lost weight before P14)
Female	$6x10^{13}$ vg/kg	6	culled - reached human endpoint (lost weight before P14)
Female	$6x10^{13}$ vg/kg	7	went missing
Female	$6x10^{13}$ vg/kg	7	culled - reached human endpoint (lost weight before P14)
Female	$6x10^{13}$ vg/kg	6	culled - reached human endpoint (lost weight before P14)
Female	$6x10^{13}$ vg/kg	8	culled - reached human endpoint (lost weight before P14)
Female	$6x10^{13}$ vg/kg	7	culled - reached human endpoint (lost weight before P14)
Female	$6x10^{13}$ vg/kg	6	went missing
Female	$6x10^{13}$ vg/kg	7	culled - reached human endpoint (lost weight before P14)
Female	$1x10^{14}$ vg/kg	6	culled - reached human endpoint (lost weight before P14)
Female	$1x10^{14}$ vg/kg	13	found dead
Female	$1x10^{14}$ vg/kg	6	culled - reached human endpoint (lost weight before P14)
Female	$1x10^{14}$ vg/kg	7	culled - reached human endpoint (lost weight before P14)
Female	$1x10^{14}$ vg/kg	7	culled - reached human endpoint (lost weight before P14)
Female	$1x10^{14}$ vg/kg	6	culled - reached human endpoint (lost weight before P14)
Female	$1x10^{14}$ vg/kg	5	found dead

Supplementary Table 2. Mean neuromuscular junction areas (in μm²) and SD for α-BuTx and nerve staining, and 2-way ANOVA with Sidak's multiple comparison test to look for gender differences.

		Mean values		SD		2-way ANOVA male vs female	
	Stain	male	female	male	female	Summary	Adjusted P Value
WT saline 1 month	BuTx	327.9	242.5	52.11	96.71	ns	0.9376
	Nerve	220.6	182	39.16	80.6	ns	0.9323
WT saline 3 months	BuTx	332.2	269.5	53.72	80.12	ns	0.9982
	Nerve	284.8	224.2	64.52	58.92	ns	0.9618
$6x10^{13}$ vg/kg 1 month	BuTx	1197	614.6	350.8	268.5	**	0.0017
	Nerve	595.6	384	93.34	109	**	0.0091
$6x10^{13}$ vg/kg 3 months	BuTx	2064	1772	865	742.8	ns	0.8123
	Nerve	804.1	671.8	334.9	201.1	ns	0.6469
$1x10^{14}$ vg/kg 1 month	BuTx	990.2	1242	235.2	169.8	ns	0.2601
	Nerve	485.2	663.4	133.5	105.2	*	0.0306
$1x10^{14}$ vg/kg 3 months	BuTx	1720	1950	282.2	636.1	ns	0.9618
	Nerve	755.7	836.7	138.2	173.7	ns	0.887

Supplementary liver toxicity study

Method

A Toxicology Study was conducted at Agilex (AU) in standard good lab practice (GLP) conditions. The design of the study was adapted from OECD Guideline for Testing of Chemicals No. 407 'Repeated Dose 28-Day Oral Toxicity Study in Rodents' 2008. Study procedures were conducted according to related test facility Standard Operating Procedures. Adult WT male and female (females were nulliparous and non-pregnant) mice were used in a 50:50 ratio and dosed accordingly to Supplementary Table 2. Animals were subjected to at least a seven-day acclimation period prior to dose administration. The acclimation period included a health examination and only animals without visible signs of illness were used for the study. Animals were identified by unique animal numbers associated with a microchip implanted subcutaneously at the time of receipt. A microchip scanner was used to read the microchip prior to all procedures.

Vehicle or AMP-101 were administered by bolus intravenous injection in a volume of 3.64 mL/kg on Study Day 1. The injection site was the lateral vein on the mouse tail and the treatment was performed with restraint on conscious animals. Tissues were collected in 10% neutral buffered formalin and forwarded to StageBio where they were processed, embedded in paraffin, sectioned, and stained with hematoxylin and eosin (H&E) for necropsy examination.

Results

To determine whether AMP-101 induces toxic side effects, a toxicology study was conducted in C57BL/6 mice. Animals were administered either Vehicle or AMP-101 up to a maximum dose of 2 x 10^{14} vg/kg via intravenous route with a single dose treatment (Supplementary Table 2). Animals were euthanized and subjected to necropsy at day 29/30 or 183/184. Tissues were processed, embedded in paraffin, sectioned, and stained with haematoxylin and eosin (H&E). All animals survived to the scheduled Day 29/30 or Day 183/184 necropsies. AMP-101 related microscopic findings at Day 29/30 and day 183/184 were not considered adverse given All the macroscopic findings observed at the day 29/30 and day 183/184 necropsies were minimal and mild, which are considered incidental and of the nature commonly observed in this age of C57BL/6 mice (including group 1 controls); therefore, they were considered not AMP-101 related (Supplementary Tables 3 and 4).

Supplementary Table 3. Dosing regimen of toxicology study

Group	AMP-101 Nominal Dose (vg/kg)	Dose Route	Dose Volume (mL/kg)	Treatment Day	Number of C57BL/6 Mice	Termination Days
1	Nil	IV	3.64	1	5 M + 5 F	29/30
					5 M + 5 F	183/184
2	6×10^{13}	IV	3.64	1	5 M + 5 F	29/30
					5 M + 5 F	183/184
3	2×10^{14}	IV	3.64	1	5 M + 5 F	29/30
					5 M + 5 F	183/184

Supplementary Table 4. Histopathological findings after 29/30 days

Liver + Gall Bladder	Male			Female		
	Group 1	Group 2	Group 3	Group 1	Group 2	Group 3
Animals per group	5	5	5	5	5	5
No visible lesions	2	0	2	1	0	0
Total Infiltrate, mononuclear cell; multifocal	2	5	3	4	5	5
of which were minimal	2	4	3	4	5	5
of which were mild	0	1	0	0	0	1
of which were severe	0	0	0	0	0	0
Total necrosis, single cell; Hepatocyte	1	4	1	0	0	1
of which were minimal	1	4	1	0	0	1
of which were mild	0	0	0	0	0	0
of which were severe	0	0	0	0	0	0
Total infiltrate, mixed cell; multifocal	1	0	0	0	0	0
of which were minimal	1	0	0	0	0	0
of which were mild	0	0	0	0	0	0
of which were severe	0	0	0	0	0	0

Supplementary Table 5. Histopathological findings after 183/184 days

Liver + Gall Bladder	Male			Female		
	Group 1	Group 2	Group 3	Group 1	Group 2	Group 3
Animals per group	5	5	5	5	5	5
No visible lesions	2	0	2	1	0	0
Total Infiltrate, mononuclear cell; multifocal	2	5	3	4	5	5
of which were minimal	2	4	3	4	5	5
of which were mild	0	1	0	0	0	1
of which were severe	0	0	0	0	0	0
Total necrosis, single cell; Hepatocyte	1	4	1	0	0	1
of which were minimal	1	4	1	0	0	1

Liver + Gall Bladder	Male			Female		
	Group 1	Group 2	Group 3	Group 1	Group 2	Group 3
Animals per group	5	5	5	5	5	5
No visible lesions	3	2	0	1	0	1
Total Infiltrate, mononuclear cell; multifocal	2	3	5	4	4	4
of which were minimal	2	3	4	2	2	2
of which were mild	0	0	1	2	2	2
of which were severe	0	0	0	0	0	0
Total necrosis, single cell; Hepatocyte	1	0	0	0	0	0
of which were minimal	1	0	0	0	0	0
of which were mild	0	0	0	0	0	0
of which were severe	0		0	0	0	0
Total infiltrate, mixed cell; multifocal	1	0	0	0	1	0
of which were minimal	1	0	0	0	1	0
of which were mild	0	0	0	0	0	0
of which were severe	0	0	0	0	0	0
