# Supplementary material for: Dose escalation pre-clinical trial of novel DOK7-AAV in mouse model of DOK7 congenital myasthenia
Source: Brain Commun. 2025 Jan 30;7(1):fcaf046. doi: 10.1093/braincomms/fcaf046 (PMC11814498; doi:10.1093/braincomms/fcaf046)
Supplement: fcaf046_Supplementary_Data [file fcaf046_supplementary_data.zip › Supplementary video legend.docx]

**Supplementary video.** First clip shows a week old uninjected DOK7^KI/KI^ model animal on the left along with a WT littermate on the right. The WT littermate can be seen moving around, while the DOK7^KI/KI^ is much smaller and can hardly move. The second clip shows a DOK7^KI/KI^ model animal three months after injection with 6x10^13^ vg/kg of AMP-101 that begins the video at the bottom right of the screen, and subsequently moves around the enclosure. It is still noticeably smaller than the other animal in the clip, which is its WT littermate, but displays normal gait and patterns of movement comparable to its littermate.
